# Recent Surveys in the Forests of Ulu Segama Malua, Sabah, Malaysia, Show That Orang-utans (*P. p. morio*) Can Be Maintained in Slightly Logged Forests

**DOI:** 10.1371/journal.pone.0011510

**Published:** 2010-07-09

**Authors:** Marc Ancrenaz, Laurentius Ambu, Indra Sunjoto, Eddie Ahmad, Kennesh Manokaran, Erik Meijaard, Isabelle Lackman

**Affiliations:** 1 Hutan, Sabah, Malaysia; 2 Sabah Wildlife Department, Wisma Muis, Sabah, Malaysia; 3 North England Zoological Society, Chester Zoo, Chester, United Kingdom; 4 Sabah Forestry Department, Sabah, Malaysia; 5 People and Nature Consulting International, Kerobokan, Bali, Indonesia; 6 Pittsburgh Zoo, Pittsburgh, Pennsylvania, United States of America; Centre National de la Recherche Scientifique, France

## Abstract

**Background:**

Today the majority of wild great ape populations are found outside of the network of protected areas in both Africa and Asia, therefore determining if these populations are able to survive in forests that are exploited for timber or other extractive uses and how this is managed, is paramount for their conservation.

**Methodology/Principal Findings:**

In 2007, the “Kinabatangan Orang-utan Conservation Project” (KOCP) conducted aerial and ground surveys of orang-utan (*Pongo pygmaeus morio*) nests in the commercial forest reserves of Ulu Segama Malua (USM) in eastern Sabah, Malaysian Borneo. Compared with previous estimates obtained in 2002, our recent data clearly shows that orang-utan populations can be maintained in forests that have been lightly and sustainably logged. However, forests that are heavily logged or subjected to fast, successive coupes that follow conventional extraction methods, exhibit a decline in orang-utan numbers which will eventually result in localized extinction (the rapid extraction of more than 100 m^3^ ha^−1^ of timber led to the crash of one of the surveyed sub-populations). Nest distribution in the forests of USM indicates that orang-utans leave areas undergoing active disturbance and take momentarily refuge in surrounding forests that are free of human activity, even if these forests are located above 500 m asl. Displaced individuals will then recolonize the old-logged areas after a period of time, depending on availability of food sources in the regenerating areas.

**Conclusion/Significance:**

These results indicate that diligent planning prior to timber extraction and the implementation of reduced-impact logging practices can potentially be compatible with great ape conservation.

## Introduction

The natural habitat of the orang-utan, the tropical forests of Sumatra and Borneo, are declining at an alarming rate as a result of human activities, such as agriculture and timber extraction. In Borneo, approximately ten percent of the remaining forests are protected for conservation, but it is doubtful that this network of protected areas alone will ensure the long-term survival of the species that leave in these forests [1], [2]. Early studies have suggested that the orang-utan was dependant on primary forests for survival and that forest exploitation and degradation was resulting in the rapid decline of the species [3], [4], [5], [6], [7], [8]. However, it is increasingly recognized that great apes (including orang-utans) can survive in low-impact and sustainably logged forests [9], [10], [11], [12], [13], [14].

Considering that more than 75% of the wild orang-utan populations in Borneo are currently found in forests that are exploited for timber [11], [15], understanding how orang-utan populations react and adapt to logging is becoming one of the major priorities for conserving the species at the landscape scale. Nevertheless, there is still a general lack of knowledge and information regarding how orang-utans respond to different intensities of timber extraction.

In 2002, our surveys in Sabah established that the commercial forest reserves of the Ulu Segama-Malua-Kuamut-Kalabakan complex were home to approximately 4,500 individuals, making it the largest unfragmented population of wild orang-utans in Malaysia [11]. These mixed lowland dipterocarp forests are located in the central part of the State and have been exploited for timber since the late 1950s [16]. Acknowledging the importance of the forests of Ulu Segama Malua (USM) for orang-utan conservation, the Sabah State government banned logging for a ten year period at the end of 2007. In 2006–2007, we conducted new aerial and ground surveys in these forests in order to monitor population trends; identify the primary proximate and ultimate factors impacting orang-utan abundance in disturbed forests; document fluctuations of orang-utan abundance in locations exposed to different logging intensities; and to propose that orang-utan conservation should be included in the forest management plan developed for this area.

In this paper we present data on the abundance and distribution of orang-utans in the forest reserves of the Ulu Segama-Malua region that were obtained during our 2006–07 surveys. We seek to determine whether the North Bornean orang-utan subspecies (*Pongo pygmaeus morio*) can maintain viable populations in sustainably and minimally logged forests, at least in the short-term? If so,does a threshold of habitat disruption and degradation exists, where maintaining a viable population becomes impossible?. We discuss some of the possible reasons behind orang-utan resilience and we also provide general recommendations for maintaining populations in forests that are exploited for timber.

## Results

### Aerial transects

In May 2007, sixteen parallel lines interspaced by about 5 km were flown over Ulu Segama Forest Reserve for a total length of 344.4 km, and eight transects interspaced by approximately 2.5 km were flown over Malua Forest Reserve totaling 140 km (Map 1). Therefore, the total survey effort of aerial surveys was roughly 6% of the entire USM area. Land below 450 m asl accounted for 78.7% and land above 600 m asl for 5.8% of our sampling. Fair forest only represented 11.5%, and it was predominantly found on steep and higher ground located in the southern and western side of Ulu Segama, bordering the DVCA, and on the top of steep hills that were not accessible to heavy logging machinery. Highly degraded forests accounted for 52% of our sample and were characterized by the complete disruption of the original canopy structure; an extreme rarity of emergent climax trees; open areas; abundance of old/recent logging roads; and the presence of invading bushes, creepers and pioneer trees belonging to Euphorbiaceae and Rubiaceae families. We applied a correction factor of F_1_ = 0.54 to aerial indexes obtained in this overdegraded habitat to take into account the increased detectability and artificially inflated aerial indexes in overdegraded forests due to canopy openness [11]. Degraded forests accounted for 11.5%, and areas of active logging for 4.4% (mostly North Ulu Segama and Malua FR). Overall, a total number of 3199 orang-utan nests were recorded. In order to investigate if orang-utan abundance depended on topographical and connectivity features, we pooled the transects in several “Sampling Units”: North Ulu Segama (NUS); Segama East; Segama Central; Segama South and West; Malua. Within each sampling unit we then investigated possible fluctuations of density resulting from habitat differences.

*North Ulu Segama (NUS): t*he forests of NUS cover roughly 12,000 ha. These forests are surrounded by oil palm plantations in the north and the Segama River in the south. They are highly degraded as a result of over-logging and fires. The last round of timber extraction was taking place in 2007 at the time of our surveys. In most areas the canopy was completely disrupted, few trees were left standing, logging roads and open areas were common, and pioneer trees such as *Macaranga spp.* dominated the landscape. Signs of active or very recent logging activities were widespread and distributed throughout the entire region. Approximately 40% of the aerial transects were flown over lands devoid of trees, considered as unsuitable habitat for orang-utans. Although orang-utan nests were found throughout the entire NUS area, they tended to be concentrated in compartments with the best forest stands or in isolated patches of trees found in the middle of over-degraded and open areas. Nests were slightly more abundant in areas with no active logging (Table 1).

**Table 1:** General results of aerial surveys in the five sampling units distinguished in the Ulu Segama Malua Forest Reserves.

Area	Transect	Length	Aerial Index	Habitat Type	Length (km)	Aerial Index	OU density (ind./km^2^)
North Ulu Segama	**(UVWXY) north**	**20.5**	**4.169**				**1.52 (0.5–4.1)**
				Overdegraded	11.4	4.77	1.90
				Active logging	10.3	3.54	1.40
Segama East	**XYZ**	**70.0**	**0.302**				**0.13 (0.04–0.39)**
	X	24.7	0.445	Overdegraded	42.5	0.294	0.12
	Y	23.8	0.252	Degraded	15.5	0.452	0.19
	Z	21.5	0.209	Active logging	2.5	0.258	0.11
				Below 450 m asl	55.2	0.302	0.12
				450–600 m asl	7.8	0.446	0.18
Segama Central	**TUVW**	**147.6**	**1.98**				**0.79 (0.29–2.16)**
	W	35.7	1.110	Overdegraded	49.5	2.14	0.85
	V	40.9	1.501	Degraded	47.1	2.44	0.97
	U	41.0	2.739	Active logging	16.1	1.05	0.43
	T	30.0	2.652	Macaranga	18.7	1.41	0.57
				Fair forest	16.2	1.76	0.71
				Below 450 m asl	124.0	1.87	0.75
				450–600 m asl	23.6	1.16	0.47
				Above 600 m asl	12.3	2.72	1.09
Segama South West	**MNOPQRS**	**92.7**	**4.47**				**1.76 (0.64–4.80)**
	S	8.3	2.590	Overdegraded	14.9	3.425	1.35
	R	12.9	2.054	Degraded	7.0	6.143	2.40
	Q	9.3	7.150	Macaranga	6.45	1.938	0.78
	P	6.4	5.469	Active logging	3.3	2.424	0.97
	O	29.0	3.931	Fair forest	25.5	5.686	2.23
	N	18.1	6.022	DVCA	14.3	4.410	1.73
	M	8.7	4.770	Below 450 m asl	35.55	2.951	1.17
				450–600 m asl	40.5	5.290	2.07
				>600 m asl	15.8	4.652	1.83
Malua	**ABCDEFGH**	**140.0**	**4.169**				**1.64 (0.58–4.52)**
	H	4.0	3.24	Overdegraded	14.2	2.510	1.00
	G	10.2	2.223	Degraded	122.5	4.488	1.76
	F	10.0	2.268	Fair forest	3.5	6.156	2.41
	E	23.7	4.641	Below 450 m asl	136.0	4.276	1.68
	D	27.8	7.262	>450 m asl	4.0	1.720	0.69
	C	30.6	5.224				
	B	24.0	3.353				
	A	9.7	7.509				

*Segama East/Central:* in eastern and central Segama, density increased from the eastern lines (ZYX pooled together: AI = 0.3 nest km^−1^) to the western lines (WVUT: AI = 2.0 nest km^−1^): t-test; t = 3.4; df = 5; p = 0.018*. The forests of eastern Segama were highly degraded and harbored very low nest densities. Nests were more abundant in steeper, higher terrains that had patches of healthier forest and were located further away from active logging activities. In the central parts of Segama, orang-utan distribution was relatively uniform. Their abundance was lower in areas with active, on-going logging activities and maximum in regenerating and healthier forests located upland (>600 m asl), where logging activities occurred over ten years ago. However densities dropped drastically in upland forests that had recently experienced intensive logging (line T: 0.47 ind.km^−2^). In the past, tall trees were used by orang-utans to cross the Bole and Kawag Rivers, but these water bodies cannot be crossed by the animals following the removal of these trees. By considering the transect lines of the same habitat located on both sides of these rivers, we investigated local differences in nest distribution and abundance. Orang-utans were more abundant on the western rather than on the eastern side of the Bole River (West: L = 22.4 km of line, 139 nests, AI = 3.10 nest km^−1^, D_ou_ = 1.23 ind. km^−2^; East: L = 23.2 km, 89 nests, AI = 1.91 nest km^−1^, D_ou_ = 0.76 ind. km^−2^), and no difference was found on either side of the Kawag river, but orang-utan density was lower within the Kawag loop, which is more difficult for the animals to access: L = 17.9 km, 62 nests, AI = 1.73 nest km^−1^, D_ou_ = 0.69 ind.km^−2^).

*Segama South/West:* Lines M and S border the south of the protected forests of DVCA in the westmost part of Segama. Part of length O was flown over DVCA and although data is presented in Table 1, it has not been included in our final analysis. Fewer orang-utan nests were identified in forests below 450 m asl (AI = 2.95 nest km^−1^) than above 450 m asl (AI = 5.23 nest km^−1^), with a difference that is near significance: U Mann and Whitney: z = −1.938: p = 0.053. The lowest nest densities in Segama S/W were recorded in lowland areas that have been highly disturbed by active and recent logging operations, and in areas highly invaded by *Macaranga sp.* Primary and old regenerating forests found in the highlands were the least disturbed habitat due to the steep slopes that are characteristic of this habitat, which limit and prevent conventional logging practices. These forests were home to the highest orang-utan densities recorded in Segama FR with about 2.1 ind. km^−2^.

*Malua FR:* Heavy logging occurred in Malua until the end of 2007, and most forests appeared degraded (43.4% of the total aerial length) or overdegraded (56.6%). Orang-utan abundance was higher in degraded (D_ou_ = 1.76 ind. km^−2^) rather than in overdegraded forests (D_ou_ = 1.00 ind. km^−2^). The highest density with about 2.4 ind. km^−2^was recorded in the forests of the “Bornean Biodiversity Conservation Plot” that appeared to be in very good condition. These forests are mature secondary forests and show a relatively diverse tree composition and structure. Orang-utan nests were more abundant on the western side of the Malua River (lines ABCD, 33.8 km, AI = 5.65 nests km^−1^, D_ou_ = 2.21 ind. km^−2^ *versus* lines CDEF, 57.9 km, AI = 3.97 nest/km, D_ou_ = 1.57 ind. km^−2^) and in the northern region (D_ou_ = 2.02 ind. km^−2^), than in the southern area (D_ou_ = 1.20 ind. km^−2^) or in the overdegraded forests of the “Wildlife Corridor” that is located in the south-eastern part of Malua (D_ou_ = 1.00 ind. km^−2^).

### Ground transects and nesting sites

Line transects were conducted for ground truthing of the aerial data and for investigating local variations of orang-utan abundance. We performed a total of 106 ground line transects (total length of 79.4 km; average length: 742 m; range: 170–1710 m) over nine expeditions throughout USM between August 2006 and June 2007 (survey effort of 0.06%): Table 2. During this time we recorded a total of 1111 orang-utan nests built in a minimum of 35 families and 65 taxa of trees (18.0% of nesting trees were not identified, adding an unknown number of families and taxa as possible nesting site): Table 3. Four tree families and 4 genera represented 62.2% and 55.2% of all nesting sites respectively. When we considered the eight families most often used for nesting, we found no significant difference between choice of tree species for nesting and family abundance in the forest (values of Wilcoxon tests are given in Table 3). However, in highly disturbed forests, orang-utans preferentially used pioneer trees like *Neolamarckia cadamba* (40.1% in Malua and 19.4% in NUS) or *Pterospermum sp.* (4.0% and 38.4%). , In the contrary *Shorea sp.* (18.2%) and mature *Macaranga sp.* (13.7%) were preferentially used as nesting sites in the less degraded habitats or in the old-logged forests of Segama. For each survey site, we classified the forest into two major classes of habitat disturbance: degraded and overdegraded. Compared to degraded forests, overdegraded forests were characterized by: more logging roads (5.5 vs 3.4 roads/km of transect, although the difference was not significant: t-test value = 1.19; df = 8; p = 0.26); a significant lower basal area (8.0 vs 16.3 m^2^/ha: t-test = −5.51; df = 80; p<0.0001*); and a significant lower tree density (142.7 vs 214.6 trees dbh>10 cm/ha: t-test = −3.85; df = 80; p = 0.0002*). Variations of orang-utan density between overdegraded and degraded habitat were tested for in each expedition where the two types of habitat were present (n = 5). Densities were significantly higher in degraded (general average of 2.23 ind. km^−2^), versus overdegraded forests (1.36 ind. km^−2^): paired-sample t-test, df = 3, t = 6.79, p = 0.007*. Ground truthing of aerial data was achieved by (1) pooling together all ground transects from different expeditions that were run in the same area, in similar habitat type where no significant difference in encounter rates were detected; and (2) comparing these with aerial orang-utan densities obtained over corresponding areas (n = 6 sites). Results given in Table 4 show a very strong correlation (r = 0.975) and no significant difference between the two data sets (paired-sample t-test: n = 6; t = 1.929; df = 5; p = 0.11).

**Table 2:** Location, main characteristics, orang-utan densities (with associated Coefficient of Variation) of all ground surveys conducted in the USM forests.

XPDC	Location	Type	LTs	Length (km)	Altitudevasl	RoadIndex	Basal Area(Nb plots)	Tree Densityper ha	Orang-utan density(CV)
1	Malua South	Deg.	5	4.515	250–450	n/a	n/a	n/a	1.36 (34.6)
		Overdeg.	7	4.294	250–450	n/a	n/a	n/a	0.69 (33.3)
2	Malua NW	Deg.	2	2.100	200–450	6.2	4.8 (2)	90	3.18 (10.7)
		Overdeg.	2	1.000	200–450	3.3	2.5 (2)	105	1.89 (69)
	Malua NE	Deg.	7	4.647	200–450	1.1	17.4 (9)	232	1.40 (29.1)
3	Segama SE	Deg.	5	4.509	300–450	7.1	15.4 (4)	172.5	1.23 (26.4)
4–5	Segama NE	Deg.	8	6.847	300–450	2.2	21.2 (7)	294	1.8 (23.3)
		Overdeg.	19	8.878	300–450	4.5	10.2 (12)	153	0.4 (34.5)
6	Segama SW	Deg.	8	9.617	350–650	3.1	11.2 (8)	161	2.1 (22)
		Overdeg.	7	6.813	350–650	9.4	9.8 (6)	107	0.7 (25)
7	Malua NW	Overdeg.	9	6.316	200–450	3.5	9.9 (8)	151	1.61 (14.5)
8	Malua NE	Overdeg.	14	11.510	200–450	8.2	8.2 (13)	163.5	2.22 (23)
9	NUS	Overdeg.	13	8.311	200–450	2.1	4.7 (11)	110	2.72 (14.7)

Legend: Deg.: degraded; Overdeg: over-degraded; asl: above sea level; n/a: not available; Nb plots: number of botanical plots; CV: coefficient of variation obtained by Distance.

**Table 3:** Percentage of utilization of the eight most common tree families and taxa used for nesting and percentage of tree abundance recorded in 69 botanical plots in three different areas: Malua, Segama and North Ulu Segama.

	Total		Malua		Segama		NUS	
*Number*	*Nests*	*Trees*	*Nests*	*Trees*	*Nests*	*Trees*	*Nests*	*Trees*
	*1111*	*1056*	*513*	*477*	*392*	*436*	*206*	*143*
**Dipterocarpaceae**	**15.1**	**27.0**	**12.4**	**29.2**	**22.4**	**29.9**	**8.1**	**11.2**
*Shorea sp.*	*11.5*	*19.6*	*8.6*	*18.5*	*18.2*	*24.8*	*6.2*	*7.7*
*Other taxa*	*3.6*	*7.4*	*3.8*	*10.7*	*4.2*	*5.1*	*1.9*	*3.5*
**Ebenaceae** (*Diospyros sp.*)	**1.3**	**2.3**	**1.3**	**2.1**	**2.0**	**2.1**	**0.0**	**3.5**
**Euphorbiaceae**	**10.0**	**10.4**	**6.7**	**10.1**	**15.2**	**9.0**	**8.6**	**16.1**
*Macaranga sp.*	*8.2*	*8.2*	*4.2*	*6.5*	*13.7*	*8.3*	*8.1*	*14.0*
*Other taxa*	*1.8*	*2.2*	*2.5*	*3.6*	*1.5*	*0.7*	*0.5*	*2.1*
**Fagaceae** (*Lithocarpus sp.*)	**4.4**	**1.9**	**1.5**	**1.7**	**10.0**	**2.8**	**1.0**	**0**
**Moraceae** (*Ficus sp.*)	**1.1**	**0.8**	**1.0**	**0.6**	**1.2**	**0.9**	**1.4**	**0.7**
**Myrtaceae** (*Eugenia sp.*)	**2.9**	**3.0**	**1.7**	**3.1**	**4.5**	**3.4**	**2.8**	**1.4**
**Rubiaceae**	**24.5**	**6.8**	**42**	**10.1**	**3.2**	**0.8**	**21.3**	**13.3**
*Neolamarckia cadamba*	*22.9*	*4.9*	*40.1*	*8.0*	*2.2*	*0.5*	*19.4*	*8.4*
*Other taxa*	*1.6*	*1.7*	*1.9*	*2.1*	*1.0*	*0.3*	*1.9*	*4.9*
**Sterculiaceae** (*Pterospermum sp.*)	**12.6**	**7.1**	**8.7**	**3.6**	**4.0**	**3.7**	**38.4**	**29.4**
**Other tree families**	**28.1**	**40.9**	**24.7**	**39.6**	**37.5**	**47.7**	**18.4**	**24.5**
Wilcoxon test values	z = −0.059; p = 0.953		Z = −0.652; p = 0.515		Z = −1.601; p = 0.109		Z = −0.059; p = 0.953	

**Table 4:** Orang-utan density estimates achieved during ground and aerial surveys over corresponding areas.

Areas	Estimated ground density	Estimated aerial density
Kawag (4–5^th^ XPDC)	1.1	0.8
Silviculture area (3^rd^ XPDC)	1.2	0.8
West Segama (6^th^ XPDC)	2.7	2.4
South Malua (1^st^ ; 7^th^ XPDC	1.1	1.2
North Malua (2^nd^; 6^th^ SPDC)	2.05	2.0
Sebagaya (4^th^ XPDC)	0.1	0.1

The total orang-utan population size living in USM was obtained by combining the knowledge gained from aerial and ground transects and by following the stratification pre-established from our aerial lines: Table 5, Fig. 2. Our final estimate is that there are 2,580 orang-utans (968–7275) in the forests of Ulu Segama Malua.

**Table 5:** Number of orang-utans living in the USM forests estimated from the combination of ground and aerial surveys (See Figure 2 for the exact locations of the areas).

Area Code	Size (km^2^)	Location	Density.	95% CI	Orang-utan Number	95% CI
1	16.24	Sepagaya	0.05	0.0–0.15	1	0–2
2	33.84	WCA	0.05	0.0–0.15	2	0–5
3	381.76	East Ulu Segama: BW 7/03 – Taliwas – west BW 7/02 BW 7/01	0.15	0.05–0.4	57	19–173
4	51.84	Central BW 7/02	0.4	0.14–1.12	21	7–58
5	98.08	North Kawag Region: Kawag loop – BW 7/03 and BW 7/04	0.7	0.25–1.92	69	25–289
6	216.16	East Bole Area	0.8	0.29–2.19	173	63–474
7	462.88	West Bole Area: Wildlife Corridor – South Malua	1.1	0.40–3.0	509	187–1387
8	150.72	South Bole Area: West BW 7/01 – East BW 7/00	1.2	0.44–3.27	181	66–493
9	187.28	South Ulu Segama: BW 7/00 – DCVA buffer	0.9	0.33–2.46	169	62–460
10	115.1	North Ulu Segama: North BW 7/04	1.5	0.55–4.02	172	84–622
11	340	South West Ulu Segama: BW 7/99 – DCVA buffer	2	0.73–5.47	680	248–1861
12	173.2	North Malua	2	0.73–5.47	346	127–948
13	50.48	West Malua: YS 3/03	1.6	0.58–4.36	81	30–220
14	56.08	South-west Malua	2	0.73–5.45	112	41–306
15	2.72	Sabah Biodiversity Plot	2.4	0.87–6.59	7	2–18
**TOTAL**					**2580**	**1295–5866**

## Discussion

It is now well established that estimating great ape abundance from nest densities can yield highly imprecise results due to the fluctuation in the nest decay rate values, amongst other factors [17]. Repeated nest counts are one way to reduce imprecision but the time required in these exercises further reduce the areas being investigated by surveyor teams [18]. In addition, monitoring the large areas that are typically occupied by great ape populations require major efforts that are difficult to match in the field due to financial, human and time constraints. Aerial surveys offer an interesting and cost-effective alternative to monitor orang-utan populations at the landscape level [11].

Combining ground and aerial surveys achieved a precise knowledge about the distribution, abundance and some of the factors influencing the orang-utan population living in the degraded forests of Ulu Segama Malua. Aerial surveys increased the general survey effort to 5.8% of the entire survey area, which is one of the highest scores documented for great apes [19] and is above the limit of 0.26%, recently proposed to achieve reliable nest abundance estimates [20]. A strong correlation was obtained between aerial and ground results, further validating the model recently developed in Sabah [11]. The discrepancy between aerial and ground indices identified in the forests of North Ulu Segama (NUS) was explained by the extreme degradation of this habitat. Because this area is a mosaic of trees left standing in bare land, ground line transects were predominately located in forested areas, while bare landscapes were typically avoided, in order to optimize time spent in the field. Therefore, orang-utan estimates were only available for forested areas and achieved a high score of 2.72 individuals/km^2^, without considering unsuitable habitat. On the contrary, aerial surveys covered all habitat types, forested or not, which resulted in an overall lower density (1.52 ind. km^−2^) compared to the ground data. Since our flights indicated that only 60% of the habitat was suitable for orang-utans, we used this stratification factor and ended up with similar population size estimates for NUS for ground (2.72×0.6×120 km^2^ = 194 orang-utans) and aerial data (1.52×120 = 182 orang-utans).

Our surveys in 2007 in the forests of USM yielded similar population estimates (2,600 individuals) to our 2002 surveys (2,300 individuals; 95% confidence intervals between 1,744 and 3,657), indicating that this population has been relatively stable over this five-year period. However, this general picture hides fluctuations that are occurring at geographical and local scales throughout the entire landscape. The uneven orang-utan abundance in USM results from the interaction of historical, man-made and natural features.

### Orang-utan abundance and human history

The scarcity of orang-utan nests identified in the eastern forests of USM (densities comprised between 0 and 0.2 ind. km^−2^) can be related to the regional human history. Eastern Sabah has been inhabited for approximately 30,000 years, based on the earliest signs of human occupation in the State [21], and small human communities have been permanently established along the lower part of Segama River for centuries [22]. During this time, people were venturing into the upper parts of Segama for hunting expeditions and to take refuge in times of trouble and epidemics [23]. Trading with China and other nations blossomed in the 15^th^ century, targeting forest products and animal parts (rhinoceros horns, nests of swiflets, hornbill skulls etc.). Hunting orang-utans for meat, traditional medicine and for skulls (after the ban of human head-hunting), might have taken its toll on the original population in this area, and possibly led to local extinction. Currently, orang-utan densities in lower Segama are at their lowest close to well-established villages [16], as has been shown for other orang-utan populations that are subjected to hunting pressure [24]. Orang-utans are slow breeders and any given population will go extinct if the yearly hunting level exceeds 1% of a particular population [25]. In addition to the probable impact of past hunting pressure, our botanical plots revealed that the eastern forests of USM were heavily disturbed, which was identified by the lack of medium and large sized trees, a low basal area, the over-abundance of pioneer tree species and the extreme rarity of sizeable dipterocarp trees and other mother trees. These findings indicate that past fires or clear-cuting during previous logging cycles have ravaged these forests and may have wiped out local orang-utan sub-populations.

### Orang-utan abundance and natural features

Orang-utan densities were higher in the west than in the east, and reached 2.0 to 2.5 ind./km^2^ in some parts of Malua and southwest Segama. However, very few nests were recorded in limestone habitats and in forests growing on ultra basic soils originating from Bidu Bidu and similar associations. The lack of sodium and the relative abundance of nickel, chromium and cobalt characteristic of these soils limits the growth of many plant species, resulting in a less diverse tree community with fewer food resources than other forest types, which accounts for the lower nest abundance in these suboptimal orang-utan habitats [26]. Large bodies of water, such as the Segama River, represent a barrier to orang-utans dispersal [27], [28]. Orang-utan abundance showed differences on both sides of the Malua, Bole and Kawag Rivers, indicating that these bodies of water may act as potential barriers for dispersal following felling of large trees that originally acted as natural bridges (Figure 2; Table 5). In Borneo, orang-utan densities usually decrease with altitude and drop sharply above 500 m asl [5], [29], [30], [31]. However in USM, high concentrations were locally recorded above this threshold in several areas (2.7 ind./km^2^ in Segama Central; 1.8 ind./km^2^ in Segama SW), while densities were significantly lower in surrounding lowland forests (Table 1). In most cases, logging activities had recently occurred or were taking place concurrently to our surveys in the surrounding lowland forests. We can therefore hypothesize that logging resulted in the forced migration into the less preferred highland forests, where orang-utan populations sought “refuge” by leaving disturbed areas [11], [29]. In large parts of southwest USM with no signs of recent logging in surrounding lowlands, valleys and ridges located above 450 m asl harbored about 2 individual km^−2^. These highland forests were lightly logged 15 years ago, but their tree diversity, size, height and canopy cover achieved the best scores among all of the survey sites, indicating that these are mature habitats or forests in a healthy regeneration stage. Oak trees belonging to the Fagaceae family (Lithocarpus spp.) were particularly common and were fruiting shortly before and during surveys. Acorns are one of the favored orang-utan foods (Russon et al, 2008), and their abundant production could have attracted animals from lower lands, possibly explaining the seasonally inflated orang-utan abundance in these hills (Singleton, 2000).

### Orang-utan abundance and logging

Overall, the habitat found in USM is very heterogeneous. This is a result of conventional logging practices and heavy extraction rates, coupled with high road densities and indiscriminate felling, that has led to a mosaic of highly degraded forests bordering hilltops and isolated patches of moderate habitat in a chaotic pattern. This condition results in extremely patchy and uneven nest distribution resulting in the large variation in nest encounter rates between ground transects located in the same survey areas, or in aerial scores fluctuating by more than 30 nests between two successive observation periods of 30 seconds (representing a distance of about 500 m). Orang-utans feed on a wide range of plants [32], but their density is limited by the frequency and duration of periods of food shortage and is correlated with fruit abundance during periods of low fruit availability [33]. Therefore, sites experiencing extreme periods of food shortages support lower population densities. In response to fruit shortages orang-utan's shift their diet to non-fruit sources and more fibrous vegetation like leaves and barks [5], and alter their range patterns in order to exploit alternative food resources [34]. In Borneo, forests dominated with dipterocarps experience extreme temporal fluctuations in fruit availability; dipterocarp abundance is negatively correlated with orang-utan abundance at many sites [14]. Sustainable and selective logging typically targets a small number of trees that are primarily Dipterocarps. After felling, timber species are replaced by pioneer and asynchronous trees (such as *Dracontomelon sp.*, *Ficus sp.*, *Neolamarckia cadamba*, etc) and light-demanding woody climbers (*Spatholobus sp.*, *Uncaria sp.*, etc). By fruiting more frequently than climax tree species and by providing young leaves and bark, these pioneer plants are supplying new and alternative food sources that buffer periods of food scarcity. In addition, exploited habitats experience changes in fruiting event patterns and species such as *Garcinia sp.* and *Litsea sp.*, which are part of the orang-utan diet, will bear more fruit during this time, providing additional resources to the animals [33].

Our results show that in USM, lightly logged forests supported relatively high orang-utan densities that were occasionally higher than those encountered in some primary lowland mature forests (see Table 1). Forests that were only logged once, over15 years ago, supported the highest orang-utan densities during our surveys, showing that orang-utans recolonize old regenerating forests and can re-establish densities similar to or even exceeding pre-logging conditions [10], [14]. Densities documented close to the Bole River during our surveys (around 2 ind./km^2^) are comparable with orang-utan abundance documented when these forests were still pristine [29], indicating that orang-utans have maintained their numbers in this area even though it has been subjected to 40 years of logging activities. However, forest patches with active disturbance systematically yielded lower orang-utan densities than surrounding forest that were not exploited at the time of our surveys, suggesting that the animals take refuge in less disturbed areas as suggested by Mac Kinnon [29]. Recolonization of previously logged areas will depend on the intensity of logging activities and the regeneration dynamic of the forest. The two most abundant pioneer trees identified during our surveys were *Macaranga sp.* (Euphorbiaceae) in Segama and *Neolamarckia cadamba* (Rubiaceae) in Malua. *Macaranga* colonizes quickly in clear-cut areas and old logging roads and has the ability to outgrow other tree species, resulting in sizeable pure stands in the most degraded areas. These trees produce wind-dispersed seeds and offer very little food resources to the fruit eating community. *N. cadamba* on the contrary, produces both fruit and bark that are edible and consumed by orang-utans. In Malua, we recorded numerous signs of bark consumption and a huge proportion of nests built in these trees (Table 3). *N. cadamba* with its spaced crown also does not restrain other trees from colonizing the areas, which helps to maintain a more bio-diverse forest within a localized area. Orang-utan density was significantly higher in areas of *N. cadamba* growth than in *Macaranga* dominated areas (1–2 individuals km^−2^ versus 0.1–0.4 ind km^−2^). Because of lower food availability in *Macaranga* dominated regions, orang-utans have to forage over a much larger area, which results in lower densities in these forests.

At all survey sites, extremely damaged habitats yielded fewer nests than lightly logged forests (Table 1). Mechanical logging inflicts structural and incidental damages to all tree size-classes [35], and heavy logging results in impoverished forest composition (fewer tree diversity, fewer food sources) and structure (lower tree density, basal area and canopy height, absence of tall trees and contiguous canopy). The destruction of fallback food sources such as *Ficus sp.* and other key plant species in overlogged areas further impoverishes the habitat and induces significant orang-utan population decline. Simplification of the forest and destruction of the original forest mosaic, force orang-utans to either use a larger range or to adopt a “sit and wait” strategy to save energy and to digest more fibrous food [36]. When food resources are destroyed over large areas, this leads to a drastic decline, as documented for the orang-utan sub-population found in the NUS area. This sub-population is completely isolated from the main population by large oil palm plantations and by the Segama River. In 2002 before the latest logging cycle, the NUS forests were already highly degraded as a result of past fires and logging activities but they were still home to approximately 400 individuals [11]. Whereas in 2007, our estimates found that there were less than 200 animals in these same forests. This decline was due to the most recent logging cycle, which left an extremely degraded habitat with acutely low tree densities and basal areas, extensive openings in the canopy and very few food resources besides the leaves and bark of pioneer plants.

Eastern Borneo suffers the most from the El Nino Southern Oscillation events and from the resulting droughts, fires and periods of food scarcity [37], [38]. As a result, orang-utans have to survive on alternative food sources such as barks and leaves for extensive periods of time. Some anatomical features of the North eastern Bornean subspecies *P.p.morio* (more robust jaw bones, thicker teeth enamel, smaller skull size) could possibly be related to the specific ecological traits of the region [39]. It has been hypothesized that these anatomical features may predispose this subspecies to cope better with habitats with fewer fruit sources that have abundant fibrous fallback foods that are typical of lightly exploited forests [14], [40].

However, the wide dietary flexibility of the species as shown by the number of plants included in their diet [32], their intimate knowledge of the forest and their faculty of storing fat when food is abundant in the forest [41] are major factors accounting for the possible adaptation of the species to some level of habitat disturbance.

### Conclusion

Our surveys in USM show that orang-utan populations can be maintained in lightly and sustainably logged forests but decline and are eventually driven to localized extinction in forests that are heavily logged or subjected to fast, successive coupes following conventional extraction methods. For example, the rapid extraction of more than 100 m^3^ ha^−1^ of timber, led to the crash of the sub-population found in the NUS forests.

Considering that the majority of great apes are currently found outside of protected areas in Africa and in Asia, it is clear that conserving these iconic species requires the establishment of a viable network of protected forests among a mosaic of certified logging concessions and other suitable habitats [13], [42]. However the orang-utan conservation community is not necessarily ready to seriously support the idea of conserving orang-utans in working timber concessions. This is partly due to the strong belief, to some extent based on empirical evidence, that all logging harms orang-utans [4], [14], and that therefore conservation of the species in timber concessions was not an option. Another factor may be that many conservationists find it difficult to entertain the notion of protecting a species in a forest that is not managed primarily for conservation purposes [43], even if orang-utans can survive in such habitats. We emphasize that with the majority of orang-utans occurring outside protected areas, and often in timber concessions, long-term protection of the species will require working closely with the timber industry and with concession managers. Not only are such programs more cost-effective nthan establishing new protected forests, they are also more likely to get support from government and other stakeholders [44].

To make orang-utan conservation in timber concession work, several crucial steps are required. The key recommendations for reconciling logging practices and orang-utan conservation, is the creation, preparation and implementation of a precise, pre-harvesting conservation management plan, in order to reduce incidental damages during felling and the adoption of reduced-impact logging practices (following the “Forest Stewardship Council” or another internationally recognized body) that will be followed during all exploitation phases. Priority will be placed on a strictly enforced, zero-hunting policy, as it has been shown that the poaching of orang-utans (or other great apes) will inevitably lead to population extinction. Crucial ecological resources (like riparian forests or patches of rich lowland forest ) need to be identified and set aside from exploitation compartments, and major food resources (like large fruit tree and key staple food plants like Ficus sp. and large climbers) must be protected from possible damages. Compartments to be exploited should follow a rotation system that allows for the creation of “refuge areas” that can be used by animals when they leave the vicinity experiencing active disturbance. Areas badly impacted by extraction activities (like stumping grounds or major logging roads) need to be rehabilitated and replanted with a mixed array of fast-growing fruit and timber trees, in order to enhance food resources in the forest. In silviculture treatment areas, large woody climbers that produce leaves and bark that are consumed by orang-utans and enable them to move throughout the forest, should not be cut. Finally, a thorough orang-utan bio-monitoring program must be developed and implemented to document population trends and their fluctuations in response to different logging regimes. If such management practices become standard practice in all remaining unprotected orang-utan habitats, chances of long-term survival of orang-utans in the wild would significantly increase.

## Materials and Methods

### Physical features of Ulu Segama Malua

**Figure 1:**
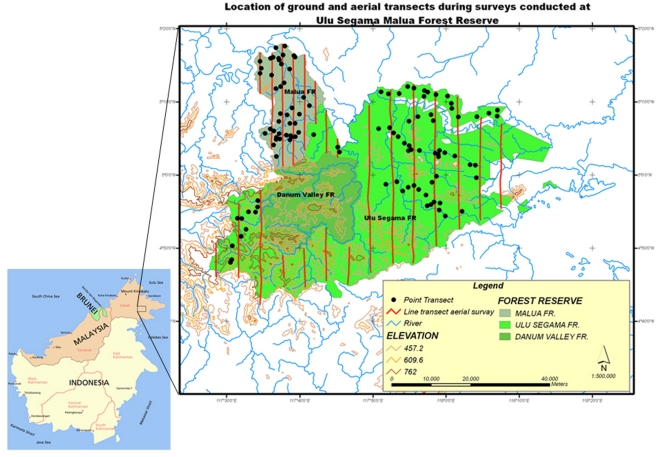
Location of aerial and ground surveys in Ulu Segama Malua, Sabah, Malaysian Borneo.

The block of Ulu Segama/Malua (USM) is located in south central Sabah between 116°28′E and 4°14′N: it comprises the commercial forest reserves of Ulu Segama (202,856 ha) and Malua (33,969 ha), as well as four protected virgin jungle forests totaling 4,273 ha (Figure 1). The USM landscape is primarily below 600m asl and consists of a variably steep terrain, with many hills and ridges that reside over the plains which are typically located close to the large rivers (Segama, Bole, Kawag). Slopes above 25% cover 10% of the entire USM system and steep hills reaching 1250 m asl are concentrated in the areas surrounding “Danum Valley Conservation Area” (DVCA). Malua's topography is predominately flat and low except for a lone hill reaching roughly 700 m asl on its western flank and the higher land located close to DVCA. Geologic formations in USM include: crystalline basement originating from metamorphic and igneous rocks of the lower Triassic which are predominantely located along rivers; Chert-Spilite sandstone from the late Cretaceous; and younger sedimentary and volcanic rocks from the formation of the Kuamut area. Most of the soils are acidic, with a pH ranging from 3.6 to 5.4, are easily eroded and lose nutrients rapidly when they are disturbed. The wet tropical climate is created by the Indo-Australian monsoon system, the average annual temperature is 27°C (with an average maximum and minimum temperature of 31°C and 23°C respectively), and rain is brought in by the northerly winds from December to March. From 1976 to 1996, annual rainfall varied between 1775 mm to 3708 mm, with regular deficits every few years (El Nino). These droughts can be severe and result in intense tree mortality and fires [45].

**Figure 2:**
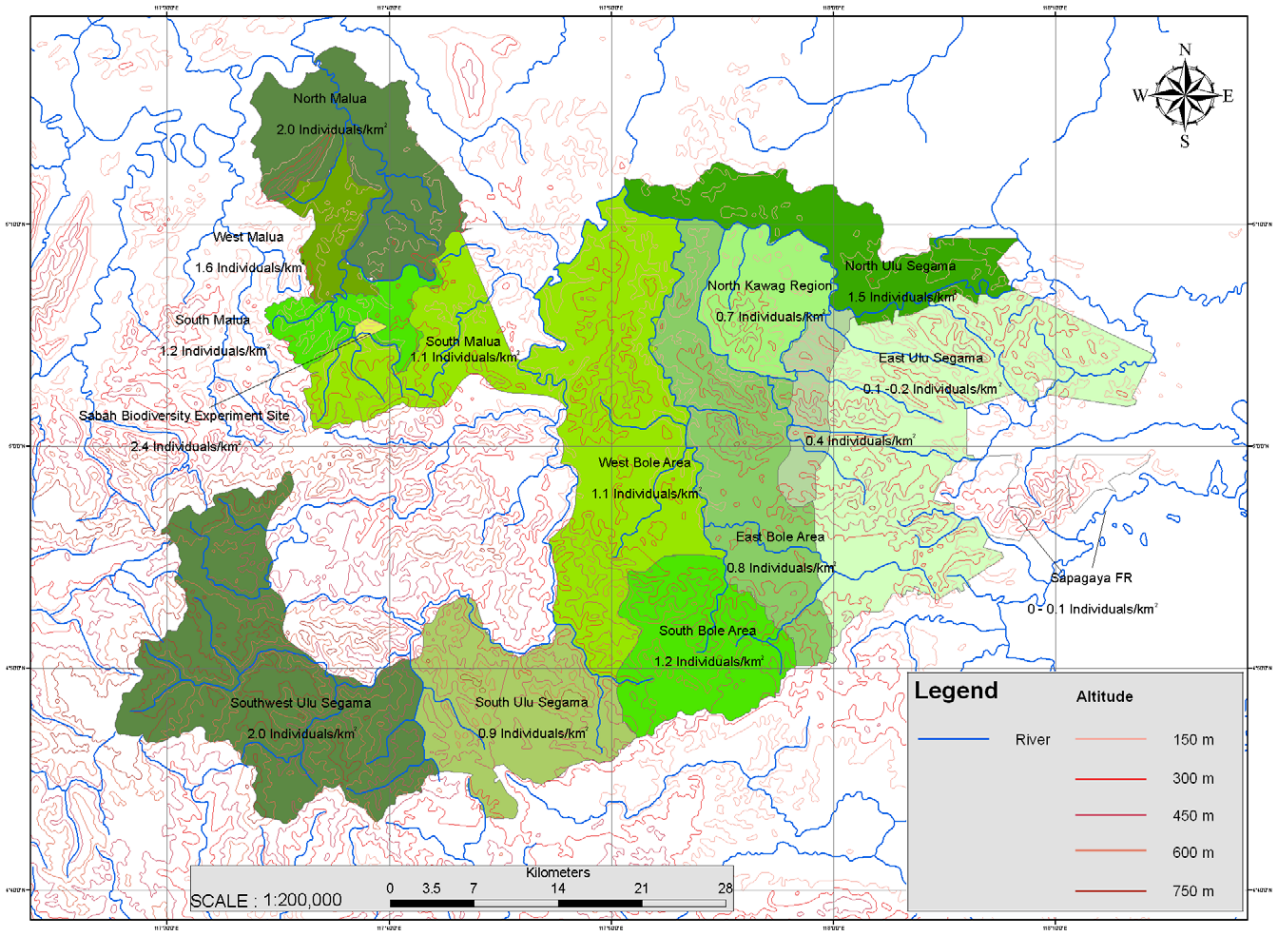
Orang-utan densities in the different areas of Ulu Segama Malua forests.

### Forest exploitation and forest types

Three major forest types occur naturally in USM and all are dominated by dipterocarp trees. The Lowland Mixed Dipterocarp Forest (LMDF) is typically found below 500 m asl. Common families include Dipterocarps (representing at least 60% of the basal area and 30% of the total tree density), Tiliaceae and Sapotaceae. Upland Mixed Dipterocarp Forests (above 500 m) are characterized by the abundance of Fagaceae (oak trees). Ultramafic Forests are found on the ultra-basic soils of the Bidu-Bidu formation that are deficient in phosphorus, potassium and calcium, are floristically less diverse and are of lower stature than other forest types. Throughout the entire landscape, the original forest structure and integrity has been altered drastically following multiple logging cycles that began in 1957 and finished at the end of 2007. The first round of logging (1957–1999), produced roughly 22 million m^3^ logs from Ulu Segama (estimated production of 87.5 m^3^/ha) and 2.5 million m^3^ logs from Malua (65.5m^3^/ha). The second round of logging, which was initiated in 1999 and completed in 2007, concentrated on approximately 105,000 ha in Ulu Segama and 20,000 ha in Malua and produced a significantly lower yield of 46.5 m^3^/ha in Ulu Segama and 33m^3^/ha in Malua [16]. Only a few protected areas escaped logging activities, namely DVCA and parts of Kawag Gibong and Sepagaya Virgin Jungle Forest Reserves. In addition, some of the logged forests were damaged by fires during major droughts induced by El Nino events. More than 5,000 ha were burned in Northern Ulu Segama (NUS) in 1983. In general, burnt areas become open land that are devoid of trees and are dominated by herbs and shrubs, with few or no signs of natural forest regeneration. However, patches of isolated, fire resistant, and regenerating pioneer species and trees from the old forest can be interspaced in this landscape [21].

The USM forests are currently classified into five different classes based on the density of trees>40 cm dbh (diameter at breast height), estimated from their crown size and visual interpretation of color aerial photographs. This approach provides a quick assessment of the potential commercial timber value of a forest [16]. The central part of USM forests (198,000 ha or 82%) are classified as very poor (i.e. less than 10 trees>40 cm dbh/ha, yielding less than 20 m^3^timber/ha), while about 23,000 (12%) ha are considered moderate to poor strata (between 10 and 30 trees 40 cm dbh/ha) and only 5% (or 13,000 ha) are considered good forest. Overall, more than 3% of USM is completely open and devoid of trees, and canopy cover is less than 30% in over 70% of USM. Low-lying and easily accessible areas have been particularly damaged by extensive and repeated coupes. These areas are characterized by: prolific signs of past human exploitation (tractor roads, stumping grounds, erosion, etc); an extremely low basal area and low tree density; a highly disrupted canopy with large forest gaps; and an abundance of invasive and pioneer plant species.

### Survey methodology

The USM surveys combined ground and aerial data collection. Ground transects were randomly located on topographical maps (1∶50 000) then ran on the ground. When possible, their direction was roughly perpendicular to large rivers, hills and major roads, in order to reduce between-transect variations and to achieve more reliable density estimates [46], [47]. Transect length was directly determined using a walking-distance measuring device: along each transect a team of two cleared a straight line-path and confirmed the bearings with a compass, while a second team of three recorded information on forest type, general level of habitat degradation and nest presence. For each nest observed, we measured the perpendicular distance from the transect and recorded size, dbh, species of the nesting tree, as well as its approximate age [19]. Botanical plots (10×50 m) were randomly selected along the transects in order to characterize forest structure and composition. In all of the plots we identified the family or taxa levels of the trees with a dbh>10cm, recorded canopy height, climbers abundance, forest type and disturbance level (degraded and overdegraded).

Aerial transects followed randomly stratified parallel lines. We used a small helicopter, type Bell 206 Jet Ranger, and followed the methodology developed in Sabah and described by [11]. Helicopter speed and height were constant at 70 km/hour and 60–80 meters above the forest canopy. The co-pilot recorded the exact location of the aircraft every 30 seconds with a GPS, and collected information on habitat types, signs of wildlife presence and human activities. Combining available historic logging information and direct observation from the aircraft, we distinguished six different habitat classes: 1. Active logging; 2. Highly degraded and recently logged forests (logging less than 2 years old, many logging roads, open canopy, no re-growth of pioneer species); 3. Degraded forests (logging activities more than two years old, signs of forest regeneration, open canopy); 4. Old logged forests (logging more than 10 years old, most places –especially old logging roads- are infested with *Macaranga spp.* or Rubiaceae trees, canopy typically closed but short); 5. Fair forest (primary forest or forest lightly logged long ago, closed and diverse canopy, presence of diverse and tall emergent trees); 6. Unsuitable orang-utan habitat (river, large open areas, etc). From the back seat, two observers looked for orang-utan nests from either side of the helicopter and relayed all sightings to a nest recorder seated between them. All visible nests were recorded in this manner and the nest recorder noted the number of nests detected by the observers per each 30-s period.

### Calculations

Ground nest densities were analyzed following line-transect analysis guidelines and were computed using the software Distance 4.2 [48]. Transformation of nest density into an orang-utan density was achieved with the formula:
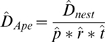
where D**_nest_** is the estimated nest density, p the estimated proportion of nest builders, t the estimated nest decay rate, and r the estimated daily rate of nest production. We used the same parameters previously determined in Sabah, to allow for direct comparison of population trends. We also used a differential nest decay rate taking into account the species of trees used for nesting [19].

Aerial indexes (number of nest per km of flight) were converted into nest and orang-utan density estimates with the model designed for Sabah and extensively described in Ancrenaz *et al.*, 2005: 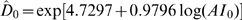, 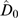 being the estimated orang-utan nest density and AI_0_ the general aerial index (AI_0_ = (AI_left_+AI_right_)/2). A final confidence interval for the predicted orang-utan nest densities was achieved with 
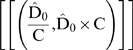
, where 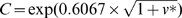 and 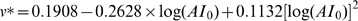. Further statistical analysis were computed with the software SPSS.
